# Infection Kinetics and Transmissibility of a Reanimated Dengue Virus Serotype 4 Identified Originally in Wild *Aedes aegypti* From Florida

**DOI:** 10.3389/fmicb.2021.734903

**Published:** 2021-09-24

**Authors:** Jasmine B. Ayers, Xuping Xie, Heather Coatsworth, Caroline J. Stephenson, Christy M. Waits, Pei-Yong Shi, Rhoel R. Dinglasan

**Affiliations:** ^1^Emerging Pathogens Institute, University of Florida, Gainesville, FL, United States; ^2^Department of Infectious Diseases and Immunology, College of Veterinary Medicine, University of Florida, Gainesville, FL, United States; ^3^Department of Biochemistry and Molecular Biology, University of Texas Medical Branch, Galveston, TX, United States; ^4^Department of Environmental and Global Health, College of Public Health and Health Professions, University of Florida, Gainesville, FL, United States; ^5^Navy Entomology Center of Excellence, Naval Air Station, Jacksonville, FL, United States

**Keywords:** *Aedes aegypti*, mosquito, infectious clone, arbovirus, dengue virus serotype 4, transovarial transmission, vertical transmission, Florida

## Abstract

Dengue virus is the most prevalent mosquito-borne virus, causing approximately 390 million infections and 25,000 deaths per year. *Aedes aegypti*, the primary mosquito vector of dengue virus, is well-established throughout the state of Florida, United States. Autochthonous transmission of dengue virus to humans in Florida has been increasing since 2009, alongside consistent importation of dengue cases. However, most cases of first infection with dengue are asymptomatic and the virus can be maintained in mosquito populations, complicating surveillance and leading to an underestimation of disease risk. Metagenomic sequencing of *A. aegypti* mosquitoes in Manatee County, Florida revealed the presence of dengue virus serotype 4 (DENV-4) genomes in mosquitoes from multiple trapping sites over 2years, in the absence of a human DENV-4 index case, and even though a locally acquired case of DENV-4 has never been reported in Florida. This finding suggested that: (i) DENV-4 may circulate among humans undetected; (ii) the virus was being maintained in the mosquito population, or (iii) the detected complete genome sequence may not represent a viable virus. This study demonstrates that an infectious clone generated from the Manatee County DENV-4 (DENV-4M) sequence is capable of infecting mammalian and insect tissue culture systems, as well as adult female *A. aegypti* mosquitoes when fed in a blood meal. However, the virus is subject to a dose dependent infection barrier in mosquitoes, and has a kinetic delay compared to a phylogenetically related wild-type (WT) control virus from a symptomatic child, DENV-4H (strain *Homo sapiens*/Haiti-0075/2015, GenBank accession MK514144.1). DENV-4M disseminates from the midgut to the ovary and saliva at 14days post-infection. Viral RNA was also detectable in the adult female offspring of DENV-4M infected mosquitoes. These results demonstrate that the virus is capable of infecting vector mosquitoes, is transmissible by bite, and is vertically transmitted, indicating a mechanism for maintenance in the environment without human-mosquito transmission. These findings suggest undetected human-mosquito transmission and/or long-term maintenance of the virus in the mosquito population is occurring in Florida, and underscore the importance of proactive surveillance for viruses in mosquitoes.

**GRAPHICAL ABSTRACT:**
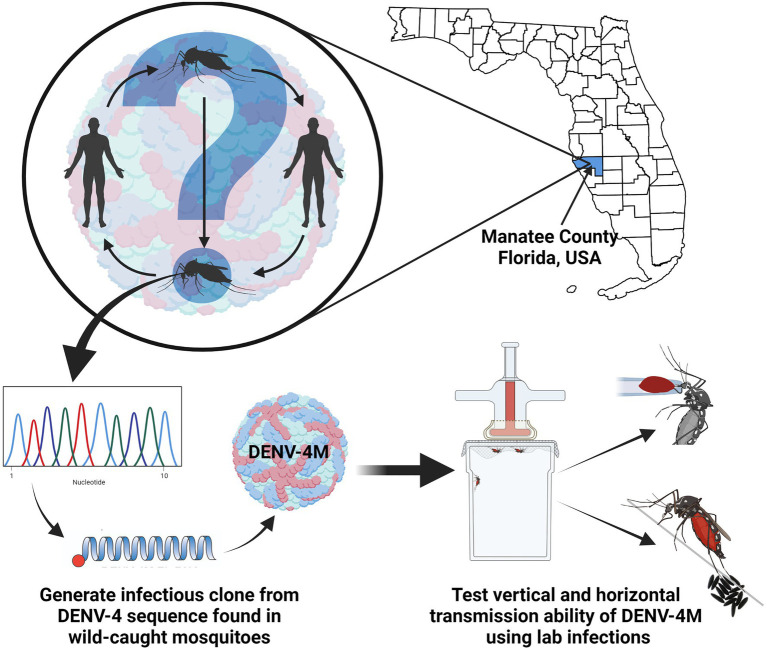
In order to better assess the public health risk posed by a detection of DENV-4 RNA in Manatee County, FL *Aedes aegypti*, we produced an infectious clone using the sequence from the wild-caught mosquitoes and characterized it *via* laboratory infections of mosquitoes and mosquito tissues.

In order to better assess the public health risk posed by a detection of DENV-4 RNA in Manatee County, FL *Aedes aegypti*, we produced an infectious clone using the sequence from the wild-caught mosquitoes and characterized it *via* laboratory infections of mosquitoes and mosquito tissues.

## Introduction

Dengue virus (DENV) is a single stranded, positive-sense RNA arthropod-borne virus in the flavivirus family, which is predominantly transmitted in human populations by the bite of infected female *Aedes aegypti* mosquitoes. DENV causes dengue fever, the most common arboviral disease in humans with approximately 40% of the world’s population at risk of infection (Scientific Working Group on Dengue. Meeting Geneva, S., and UNDP/World Bank/WHO Special Programme for Research and Training in Tropical Diseases, 2007). Four antigenically distinct serotypes of DENV circulate in human populations (denoted DENV-1 through DENV-4). Primary DENV infections are typically asymptomatic or cause non-descript, febrile illness, but subsequent re-infection with a different serotype can cause severe dengue, including dengue shock syndrome, dengue hemorrhagic fever, or death (Guzman and Vazquez, 2010). *Aedes aegypti* and a secondary dengue vector *Aedes albopictus* are widespread in Florida, United States (Reiskind and Lounibos, 2013). Imported (i.e., travel-related) cases of dengue occur in Florida every year, and locally acquired cases have been increasing over the past decade with 73 locally acquired cases in the state in 2020 alone (Mosquito-Borne Disease Surveillance, 2021). Travel associated cases of all four serotypes of DENV have been reported in Florida, but no locally acquired cases of DENV-4 have ever been reported (Mosquito-Borne Disease Surveillance, 2021).

In 2016 and 2017, we detected and subsequently sequenced the complete genome of dengue virus serotype 4 (DENV-4) from pools of field-derived F1 *Aedes aegypti* mosquitoes collected in Manatee County, Florida (Boyles et al., 2020). The presence of DENV-4 in mosquitoes in two consecutive years from the same oviposition traps without a locally acquired or travel associated human index case in the county was peculiar, suggesting either undetected human-to-mosquito transmission and/or prolonged maintenance of the virus *via* vertical transmission from mosquitoes to their progeny. Natural vertical transmission in *Aedes* has been reported for all four DENV serotypes, although its contribution to epidemic disease in humans is unclear (Ferreira-de-Lima and Lima-Camara, 2018). However, the discovery of DENV-4 from Manatee County field-derived mosquitoes was unexpected since the project was focused on characterizing the adult female *A. aegypti* microbiome and metavirome and targeting insect-specific RNA viruses in a county assumed to be well-outside the DENV infection foci of South Florida. The mosquito samples were processed solely for RNA extraction, library assembly, and subsequent sequencing, and as such, arbovirus isolation was not possible due to viral inactivation during the sample preparation process.

We generated an infectious clone virus to complete the rigorous validation of the reported viral genome sequenced from the Manatee *A. aegypti* using validated methods to produce flavivirus infectious clones (Shi et al., 2002; Zou et al., 2015; Shan et al., 2016; Xia et al., 2018). The clone was based on the published virus genome (Accession Number: MN192436.1) to characterize the viability of the resulting virus DENV-4M. Herein, we demonstrate that the DENV-4M infectious clone is indeed a viable virus, which is infectious to both susceptible mammalian and mosquito cell lines. Importantly, we observed that the infectious clone can infect *Ae. aegypti* Orlando (ORL) strain mosquitoes *per os* and is secreted into saliva at 14days post-infection. The virus also infects the ovary 14days following *per os* infection and can undergo vertical and transstadial transmission. This suggests that the initial identification of DENV-4M genomes in Manatee County mosquitoes represented a real detection of DENV-4, which changes our understanding of the public health risk of this serotype in the state. Given the possible risk of transmission to humans by bite as well as long-term maintenance in the mosquito population by vertical transmission, these data further underscore the importance of pro-active surveillance for DENV and other arboviruses in vector mosquito populations in advance of the “mosquito season” in Florida.

## Materials and Methods

### Construction of DENV-4M Infectious Clone

Four fragments (FI-FIV) spanning the entire genome of DENV-4M (GenBank accession:MN192436.1) were initially synthesized and cloned into pUC57 vector by Genscript (Piscataway, NJ). The sequences of the oligonucleotides are included in Supplementary Data Sheet 1. A T7 promoter and a hepatitis delta virus ribozyme (HDVr) sequence were engineered at the 5' and 3' ends of the fragments FI and FIV, respectively. The individual fragment was amplified by PCR using the Platinum SuperFi II DNA Polymerase (ThermoFisher Scientific, Waltham, MA, United States) with corresponding primer pairs listed in Supplementary Table S2. The resulting amplicons were assembled into a full-length clone in a single-copy vector pCC1BAC (Epicentre) by using the NEBuilder HiFi DNA Assembly kit (New England Biolabs, Ipswich, MA, United States). The cDNA sequence of DENV-4M in the full-length clone was finally validated by Sanger sequencing using the primers listed in Supplementary Table S2.

### RNA *in vitro* Transcription, Electroporation, and Virus Rescue

Full-length DENV-4M RNAs were *in vitro* transcribed using a T7 mMessage mMachine kit (ThermoFisher Scientific, Waltham, MA, United States) from cDNA plasmids as linearized by ClaI. The RNA transcripts (10μg) were electroporated into baby hamster kidney fibroblast (BHK-21) cells following a protocol described previously (Xie et al., 2011) with some modifications. Briefly, 8×10^6^ cells were suspended in 800μl Ingenio^®^ Electroporation Solution (Mirus Bio, Madison, WI, United States) and mixed with 10μg RNA in a 4-mm cuvette. Electroporation was performed using the GenePulser apparatus (Bio-Rad) by three pulses with 3s intervals at instrumental settings of 0.85kv at 25μF. After a 10-min recovery at room temperature, transfected cells were transferred into a T-75 flask containing 15ml culture media. Alternatively, 1×10^4^ transfected cells were seeded into each well of 8-well Lab-Tek^™^ II chamber slides (ThermoFisher Scientific) for immunostaining analysis. After incubation at 37°C with 5% CO_2_ for 24h, the culture medium was replenished with medium contanining 2% fetal bovine serum (FBS). The cells were then incubated at 30°C with 5% CO_2_ for an additional 4days. Supernatants were clarified by centrifuging at 1,000g for 5min at 4°C and stored at −80°C prior to use.

### Cell and Virus Culture

Baby hamster kidney fibroblast-21 cells (Xie et al., 2011) and Vero E6 cells (ATCC# C1008) were obtained directly from Pei-Yong Shi’s group at UTMB. Vero E6 cells were maintained at 37°C and 5% CO2 in complete Dulbecco’s Modified Eagle Medium (DMEM) (ThermoFisher, 11965092) supplemented with 10% heat inactivated FBS, 1 × penicillin/streptomycin (ThermoFisher, 15140122), and 1 × L-glutamine (ThermoFisher, 25030081). *Aedes albopictus* C6/36 (ATCC# CRL-1660) and *A. aegypti* Aag2 (ATCC# CCL-125) insect cell lines were obtained from ATCC and maintained at 30°C and 5% CO2 in complete Minimum Essential Medium (MEM) (ThermoFisher, 12360-038) supplemented with 10% heat inactivated FBS, 1x penicillin/streptomycin, and 1x L-glutamine. The DENV-4M was obtained from UTMB as passage 0 infectious virus at 4×10^3^ PFU/ml in Vero E6 cell culture supernatant and was used directly in experiments as described. A DENV-4 strain isolated from a symptomatic child in Haiti in 2015 (DENV-4H, strain Homo sapiens/Haiti-0075/2015, GenBank accession MK514144.1) was used as a positive control.

### *In vitro* Infection

Cells were infected at 80% confluency at 0.01 multiplicity of infection in media (DMEM or MEM as described in cell and virus culture section) containing 3% FBS (i.e., reduced serum media). After a 1-h infection period, inoculum was removed, the monolayer was washed once with 1× PBS, and fresh reduced serum media were added. For reverse transcription quantitative PCR (rt-qPCR) experiments, supernatant samples were taken at this point [0 days post infection (dpi)]. Mock infected controls were seeded and treated identically, with sterile media used instead of virus stock. To passage virus, 200μl of 5 dpi culture supernatant from the previous passage’s infected flask was used as the virus stock for the new flask. Supernatant samples were collected during passages 1, 2, 5, and 10. All cell culture experiments were performed in triplicate and each independent replicate was a culture slide well or flask processed in parallel.

### Mosquito Rearing and *in vivo* Infection

Orlando strain mosquitoes were initially obtained as adults from the Gainesville United States Department of Agriculture Center for Medical, Agricultural, and Veterinary Entomology colony. Offspring of these mosquitoes were used for experiments. Adults were maintained on 10% sucrose solution *ad libitum* at 28°C and 80% humidity with a 12:12 light:dark cycle. Larvae were reared on ground TetraMin flakes. Adult female mosquitoes were starved overnight and fed a 2:2:1 mixture of O^+^ human red blood cells (Lifesouth Community Blood Centers, Gainesville, FL, United States): infected or uninfected (in naive blood control conditions) Vero E6 cell culture supernatant: heat inactivated human serum *via* an artificial membrane feeder held at 37°C and affixed with pork sausage casing. After blood feeding, mosquitoes were cold anesthetized and non-blood engorged individuals were discarded. All remaining mosquitoes were maintained on 10% sucrose solution and given access to an oviposition surface. At 7 and 14 dpi, mosquitoes were cold anaesthetized, surface sterilized in 70% ethanol, and rinsed twice in 1 × PBS before midguts and ovaries were dissected out in 1 × PBS. In the vertical transmission experiment, mosquitoes were offered a second naive blood meal at 13 dpi, and allowed to oviposit again for 72h on new damp filter paper. These second blood feed eggs were dried completely, hatched, and reared to adulthood (as described above). Resultant adult females were surface sterilized and pooled by rearing container in pools of up to 25 (Supplementary Table S1). Each replicate (two or three replicates as indicated in figure legends) used mosquitoes from a different egg laying date, which were reared, fed, and processed separately. All mosquito infections and handling of infected mosquitoes took place in an ACL-3/BSL-2 facility.

### Salivation Assay

At 14 dpi, DENV-4 infected mosquitoes were starved overnight. To collect saliva, mosquitoes were cold-anesthetized, and their wings and legs were removed. Each mosquito was then fastened to a glass microscope slide with tape, and their proboscis was inserted into a graduated glass capillary tube (Drummond, Broomall, PA, United States) filled with 3μl of warmed human O^+^ blood (1,1 O^+^ human red blood cells: heat inactivated human serum) to initiate feeding cues and facilitate saliva collection (Stephenson et al., 2021). The mosquitoes were placed in a lit rearing chamber at 28°C with 80% relative humidity for 45min or until they ingested approximately 2μl of blood. Each proboscis was then removed from its capillary tube, and the remaining blood from each capillary tube was aspirated into 1.5ml microcentrifuge tubes with 200μl of reduced (3% FBS) DMEM. Mosquitoes were then surface sterilized in 70% ethanol and rinsed twice in 1x PBS before being dissected in PBS to produce paired ovary and midgut samples. All samples were immediately stored at −80°C until use.

### RNA Extraction and Reverse Transcription Quantitative PCR Virus Detection

At the time of collection, each tissue was placed in a 1.5ml microcentrifuge tube with 700μl chilled, sterile PBS and 0.2ml of sterile glass beads. Each tissue sample was loaded into a Bullet Blender and homogenized by running the Bullet Blender at speed 8 for 5min. Saliva, supernatant, and tissue samples were then spun down in a bench-top centrifuge at 3750 × g for 3min. Lysis buffer (560μl) AVL (Qiagen) was aliquoted into pre-labelled sterile 1.5ml microcentrifuge tubes. About 140μl of sample homogenate was aliquoted into the corresponding lysis tube. Cell supernatant samples were added directly into lysis buffer. RNA extraction on each sample was carried out using the QIAmp Viral RNA extraction kit (Qiagen) following the manufacturer’s protocols. Sample RNA was tested for DENV-4 pre-membrane protein gene (primer and probe sequences are provided in Supplementary Table S3). Each sample was run as technical duplicates, and each plate included a no template control, and a DENV-4 positive control (NR-50533, BEI resources and diluted 1:10 with nuclease-free water). Sample RNA was run with either QuantaBio UltraPlex 1-Step ToughMix (4X) Low-ROX master mix, or SuperScript^™^ III Platinum^™^ One-Step qRT-PCR on a BioRad CFX96 Touch Real-Time PCR Detection System at 50°C for 30min (for Superscript reactions) or 50°C for 10min (QuantaBio reactions), 95°C for 2min, and 45 cycles of: 95°C for 15s, and 60°C for 45s.

The rt-qPCR cycle threshold (CT) values were converted to PFU equivalents (PFUe) with a standard curve created using eight 10-fold dilutions of RNA extracted from DENV-4M Vero E6 P2 stock virus of known titer (7×10^6^ PFU/ml), run as technical duplicates, and fitted with a logarithmic line of best fit in Microsoft Excel (Version 2,105) [(*CT value*)=−1.542ln(*PFUe*)+39.355, *R*^2^=0.9905]. The limit of detection (LOD) for this assay was a CT value of 40 or 0.65 PFUe/ml.

### Immunofluorescence Assays

Unless otherwise indicated, all incubation steps were performed at 4°C in the dark, and all buffers were kept ice cold. Media were washed off of 4 dpi cells, and 7 or 14 dpi midguts or ovaries with 1x PBS. Tissues were fixed by adding 1ml 4% paraformaldehyde (PFA) in PBS+0.05% Tween20 (PBST) and leaving cells at room temperature for 10min. PFA/PBST mixture was removed, and tissue was washed 3×5min with 1ml of PBST. Tissues were permeabilized by adding 1ml 0.5% TritonX-100/PBST for 20min. TritonX-100/PBST mixture was removed and tissue was washed 3×5min with 1ml of PBST. Tissues were blocked in 1ml of 5% heat-inactivated FBS in PBST for 30min at room temperature. FBS/PBST mixture was removed and tissues were washed 3×5min with 1ml PBST. Primary antibody (200μl) in PBS was added [1:2,000 dilution of pan-serotype DENV NS1 mAb (R&D Biosystems# MAB94442-100) in 1× PBS] and tissues were incubated in a humidity chamber overnight at 4°C. Primary antibody was removed and tissues were washed 3×5min with 1ml of PBST. Secondary antibody (200μl) was added [1:1,000 dilution of Alexa Fluor^™^ goat anti-mouse 594 IgG (H+L; Invitrogen, A11005, Lot 1937185) in 1 × PBS] and tissues were incubated for 1h at 4°C in the dark. Cells were washed 3×5min with 1ml of PBST. 4,6-diamidino-2-phenylindole (DAPI) stain was added (1:200 dilution of Roche Diagnostics, Ref 10236276001, and Lot 70317525 in 1 × PBS) and tissues were incubated for 10min at room temperature in the dark. Tissues were rinsed 2x with PBST and washed once for 5min with PBS. Slides were mounted with VECTASHIELD® Antifade Mounting Media with DAPI (Vector Laboratories, Ref H-1200, Lot ZE0815) and imaged on a KEYENCE BZ-X800 microscope. Capture and image processing settings were kept constant between conditions within each timepoint/tissue.

### Plaque Assay

Supernatant samples used as virus stocks to infect cells or mosquitoes were subject to titration by plaque assay and were mixed with 10% final concentration trehalose to stabilize virions for freezing and stored in liquid nitrogen. BHK-21 cells were grown to confluency with DMEM supplemented with 10% FBS, 1% L-glutamine, 1x penicillin/streptomycin, and 0.25μg/ml amphotericin B and then seeded into 24-well plate and incubated for 2days at 37°C and 5% CO_2_. Next, each supernatant sample was serially diluted 10-fold in reduced DMEM (3% FBS). The spent media were removed from the BHK-21 cells in the 24-well plates and 100μl of each dilution series was added to individual wells. The 24-well plates were then rocked at room temperature for 15min and incubated at 37°C and 5% CO_2_ for 45min. Afterward, 500μl of 0.8% w/v methyl cellulose in DMEM containing 2% FBS was added, and the plates were re-incubated at 37°C and 5% CO2 for 5days. On the fifth day, the spent media were removed from each well of the 24-well plates, and a 1:1 methanol/acetone solution with 1% crystal violet was added for at least 1h to fix and stain the cells. The plates were then washed with water, and subsequently stored upside down overnight to drain and dry them. Plaques were manually counted, and titer expressed as plaque forming units/ml (PFU/ml).

## Results

### Construction of the Manatee DENV-4 Infectious Clone

**Figure 1:**
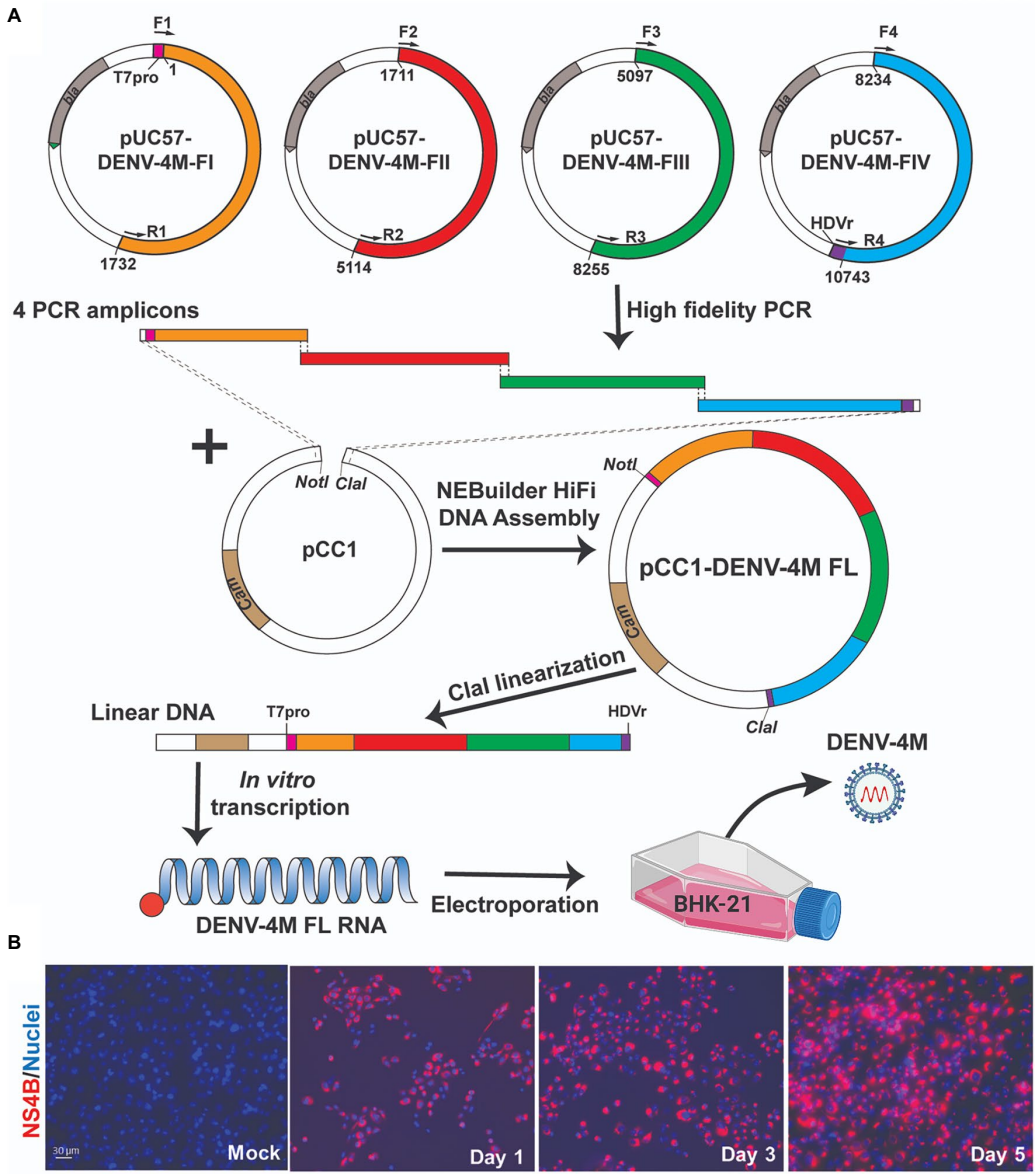
Manatee County DENV-4 (DENV-4M) parental infection data show active replication in mammalian cell culture. **(A)** Diagram of construction of DENV-4M infectious clone and generation of recombinant viruses. **(B)** Immunofluorescence assay (IFA) analysis of baby hamster kidney fibroblast (BHK-21) cells transfected with *in vitro* transcribed viral RNA. On days 1, 3, and 5 after transfection, cells were assayed by immunofluorescence for DENV nonstructural protein 4B (NS4B; red). Nuclei are stained with DAPI (blue).

Construction of full-length infectious cDNA clones of flaviviruses remains challenging due to the instability of the cDNA of the genome during plasmid propagation in the *Escherichia coli* system. We took two steps to overcome this issue. Firstly, to quickly obtain the subclones prior to assembly of the full-length infectious clone, we divided the entire DENV-4M cDNA into four consecutive fragments and cloned them into a high-copy plasmid pUC57 (Figure 1A). To enable the *in vitro* transcription of a 5' capped genome-length RNA, a T7 promoter, and a HDVr sequence was engineered upstream of the 5' untranslated region (UTR) and downstream of the 3' UTR, respectively. Secondly, upon assembly, we used high-fidelity PCR to obtain each fragment, and took advantage of the NEBuilder HiFi DNA Assembly technique to clone the four PCR amplicons into a single-copy vector pCC1BAC to increase the stability of the cDNA plasmids when propagated in *E. coli*. Nineteen- to 26-basepair (bp) overlaps were introduced into adjacent fragments. In addition, the 5' end of PCR fragment FI and the 3' end of PCR fragment FIV contain a 22-bp overlap with the region upstream of the restriction site *NotI* and a 21-bp overlap with the region downstream of the restriction site *ClaI* in the pCC1BAC vector, respectively (Figure 1A). The four fragments were then directionally assembled into the pCC1BAC that was pre-linearized by NotI and ClaI, resulting in the full-length infectious clone pCC1-DENV-4M FL.

To recover recombinant DENV-4M from the infectious clone pCC1-DENV-4M FL, we electroporated the *in vitro* transcribed genome-length RNA into BHK-21 cells. After electroporation, the intracellular expression of nonstructural protein 4b (NS4B) was examined by immunofluorescence assay (IFA). NS4B-positive cells increased from days 1 to 5 post-electroporation (Figure 1B). These data demonstrated that DENV-4M is rescued from the infectious clone and the resulting recombinant DENV-4M virus can replicate and spread on BHK-21 cells. The virus was expanded once on Vero E6 cells and then utilized for the experiments described in the remainder of the study.

### *In vitro* Immunofluorescence Assay Shows DENV-4M Replicates in Mammalian and Insect Cell Lines

**Figure 2:**
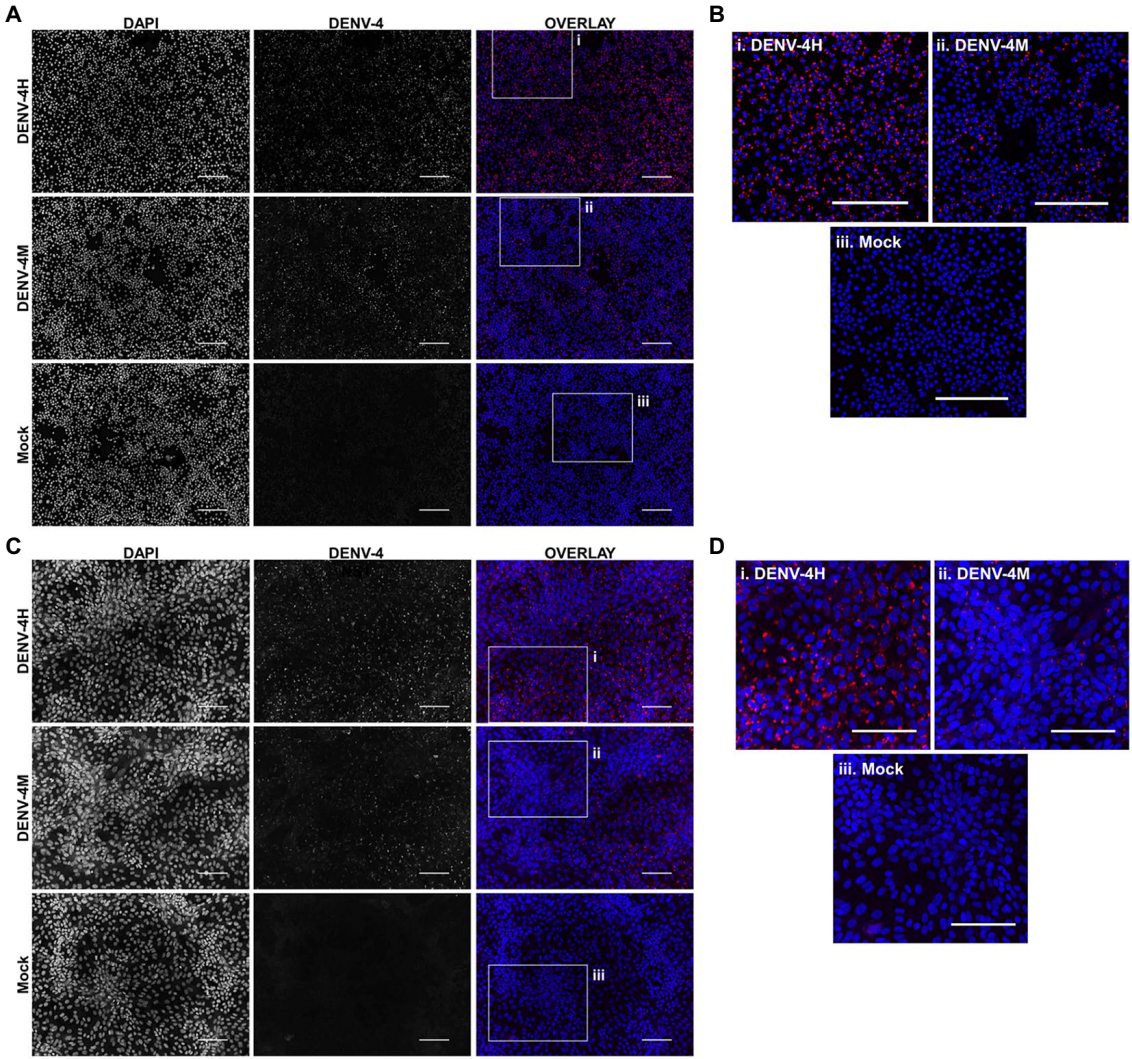
Dengue virus serotype 4 (DENV-4) nonstructural protein 1 (NS1) IFA shows viral replication in *Aedes albopictus* C6/36 cells and in mammalian Vero E6 cells. DENV-4M or DENV-4H were used to infect C6/36 **(A,B)** and Vero E6 cells **(C,D)** at an MOI of 0.01, and cells were processed for NS1 IFA at 4days post infection (dpi). Red (center column) indicates DENV-4 NS1 and blue (left column) indicates DAPI DNA counterstain. Representative images were chosen from three independent experiments. Scale bar=100μm.

To assess the viability of DENV-4M in insect tissues *in vitro*, an *A. albopictus* embryonic cell line known to be highly permissive to DENV-4 infection (C6/36) was chosen as a model insect line. African green monkey kidney cells (Vero E6) were used as a positive control. IFA was performed for DENV-4 nonstructural protein 1 (NS1) to visualize viral replication in cell cultures. DENV-4M produced NS1 signal by 4 dpi in both C6/36 (Figures 2A,B) and Vero E6 cells (Figures 2C,D). A DENV-4 strain isolated in 2015 from a symptomatic child in Haiti (DENV-4H, strain *Homo sapiens*/Haiti-0075/2015, GenBank accession MK514144.1) was used throughout the study as a positive infection control, as it is known to infect *Ae. aegypti* robustly (Stephenson et al., 2021). In both cell lines, DENV-4M showed noticeably lower infection prevalence and intensity compared to DENV-4H (Figure 2).

### Replication Rate in Insect Cell Lines Can Be Improved by Serial Passage

**Figure 3:**
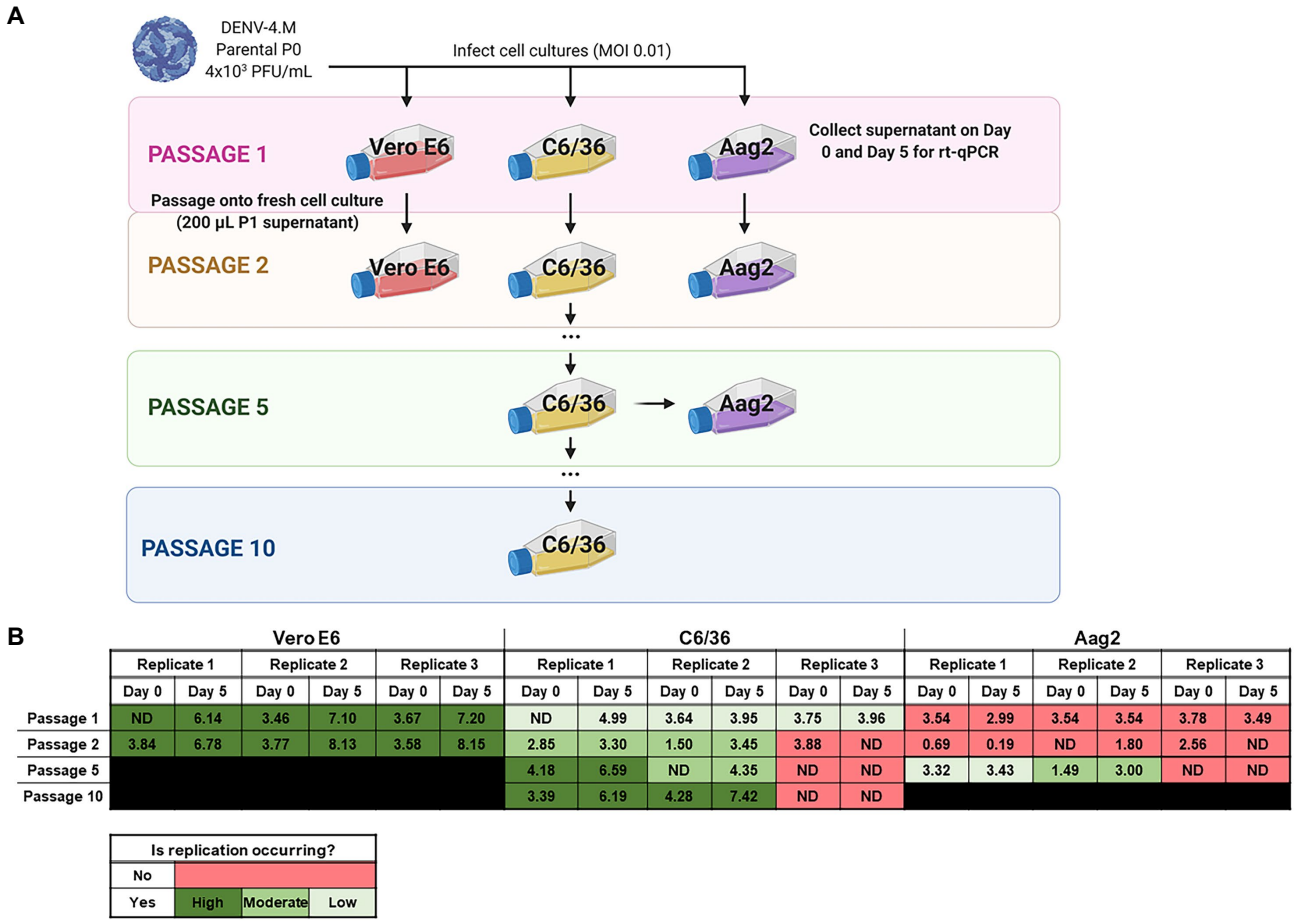
Detection of Manatee IC replication in mammalian and insect cells by reverse transcription quantitative PCR (rt-qPCR) on culture supernatant shows DENV-4M completes replication in insect cell lines and can be adapted to insect cell culture by serial passage. **(A)** Workflow schematic of serial passage experiment. One mammalian (Vero E6) and two mosquito (C6/36 and Aag2) cell lines were infected with parental (P0) DENV-4M stock virus. Cell culture supernatant was taken at day 0 (immediately following removal of inoculum) and day 5 post infection for rt-qPCR. On 5 dpi, supernatant from the infected culture was serially passaged onto an uninfected culture. Additionally, C6/36 P5 virus was used to infect Aag2 cell culture to test if adaptation to an insect cell line improves the performance of the virus in Aag2 cells. **(B)** Log_10_ DENV-4 PFU equivalents (PFUe)/ml, as determined by converting cycle threshold values with a standard curve made using 10-fold dilutions of RNA extracted from virus stock with a known PFU of 7 × 10^6^ are reported for the indicated conditions. The values reported represent the mean of two technical rt-qPCR replicates. ND indicates that no viral genome was detected in the indicated condition. The limit of detection (LOD) of the assay was a cycle threshold value of 40 or 0.65 PFUe/ml The three biological replicates reported were performed in separate tissue culture flasks processed in parallel.

To quantify the replication rate of DENV-4M *in vitro*, we performed rt-qPCR on RNA from culture supernatant collected immediately after inoculum was washed off cells (0 dpi), and on supernatant collected 5 dpi. To confirm that viable virus was being produced, we passaged supernatant from passage 1 (P1) flasks into fresh cultures and repeated the rt-qPCR quantification (Figure 3A). By this measure, DENV-4M replicated robustly in Vero E6 cells with an average 10^3.95^ (8,947-fold) increase in PFU equivalents (PFUe)/ml from 0 to 5 dpi in P2 cultures (Figure 3B). However, replication in C6/36 cells was much more modest with a maximum PFUe/ml increase of 10^1.95^ (89-fold) in the P2 cultures, with one of three replicates not producing viable progeny virus in P1 (Figure 3B). A third cell line, the *Ae. aegypti* larval cell line Aag2, did not demonstrate any replication in P1 or P2 (Figure 3B). Despite being a more relevant model (in that they are derived from *Ae. aegypti*), Aag2 cells were expected to be less permissive to replication than C6/36 cells because they have an intact RNA interference pathway while C6/36 cells do not (Brackney et al., 2010). Insect cell lines are commonly persistently infected by cell-fusing agent virus (CFAV), an insect specific *Flavivirus* which has been observed to both positively and negatively impact dengue replication in different infection models (Zhang et al., 2017; Baidaliuk et al., 2019; Fredericks et al., 2019). Both our Aag2 and C3/36 cells test positive for CFAV, so differing infection status is not a potential explanation for their differential susceptibility to DENV-4M, although persistent infection with several other insect specific viruses has been reported for both cell lines and may be a confounding factor (Weger-Lucarelli et al., 2018). Our mosquito colony discussed in later sections tests negative for CFAV, demonstrating that infection with CFAV is not required for DENV-4M to infect mosquito tissues.

As the virus replicated more robustly in P2 than P1 on C6/36 cells in two of three replicates, we continued to serially passage infected supernatant on C6/36 cells to adapt the virus to insect cell culture. At P5 and P10, samples of days 0 and 5 supernatant were retained for rt-qPCR analysis; DENV-4M replicated much more rapidly in C6/36 cells by P10 with a maximum PFUe/ml increase of 10^3.13^ (1,363-fold), comparable to its initial replication rate in Vero E6 cells (Figure 3B). DENV-4M transferred to Aag2 cells after P5 on C6/36 cells also fared better than the P0 parental stock, with two of three replicates showing replication and a maximum PFUe/ml increase of 10^1.51^ (32-fold). The ability of DENV-4M to establish replication in insect cells over serial passage was sporadic compared to Vero E6 cells, with one of three replicates in both C6/36 and Aag2 cells not demonstrating replication in cell cultures after P1 (Figure 3B).

### DENV-4M (Vero E6 P2) Is Detectable by Rt-qPCR *in vivo* and Is Capable of Horizontal and Vertical Transmission

To obtain an *in vivo* measure of viral replication, we initially fed an infectious blood meal containing parental P0 DENV-4M to adult female ORL strain mosquitoes and dissected midguts at 14 dpi, however, no midguts were found to be positive (0/43) for DENV-4 by this measure.

**Figure 4:**
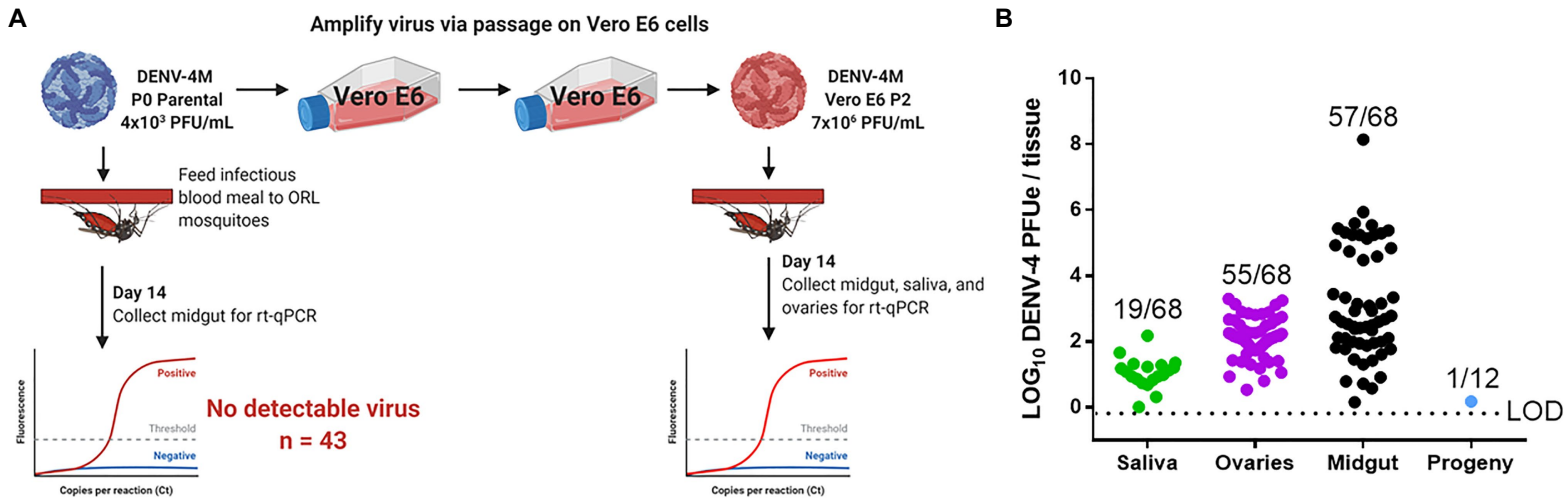
Detection of DENV-4M genome by rt-qPCR after *per os* infection in 14 dpi Orlando (ORL) mosquito tissues. **(A)** Workflow of *in vivo* infection. Parental P0 DENV-4M stock yielded no infection in 43 tested 14 dpi midguts. Virus was passaged 2x in Vero E6 cells and ORL mosquitoes were infected with this product. **(B)** Rt-qPCR was performed on saliva, ovaries, and midguts dissected from each of 68 individual mosquitoes. Log_10_ DENV-4 PFU equivalents (PFUe)/tissue, as determined by converting cycle threshold (CT) values with a standard curve made using 10-fold dilutions of RNA extracted from virus stock with a known PFU/ml of 7×10^6^ are reported. The values reported represent the mean of two technical rt-qPCR replicates. The LOD of the assay was a CT value of 40 or 0.65 PFUe/tissue. The three biological replicates reported were performed in separate tissue culture flasks processed in parallel. Only samples with a detectable CT value in both technical replicates are shown on the graph; the proportion of rt-qPCR positive samples / total samples tested is displayed over each tissue type. Results are pooled from three independent experiments (saliva, ovaries, and midgut) or two independent experiments (adult female progeny).

Based on the weak but detectable replication observed in the initial infection of C6/36 cells and the low titer of parental DENV-4M (4×10^3^ PFU/ml), we hypothesized that the resistance to infection observed in ORL mosquitoes may be dose-dependent. To test this, we fed adult female ORL mosquitoes an infectious blood meal containing DENV-4M from P2 on Vero E6 cells (7×10^6^ PFU/ml; experimental workflow illustrated in Figure 4A). DENV-4M is clearly capable of replicating in ORL mosquitoes following two passages on Vero E6 cells (Figure 4B). Midguts were collected from these mosquitoes as a proxy for infection, saliva was collected as a proxy for horizontal transmission, and ovaries were used as a proxy for vertical transmission. Infected mosquitoes showed a high infection intensity and prevalence in the midgut and ovary on day 14 (Figure 4B). Of the tested mosquitoes, 19/68 (27.9%) had detectable viral genomes in the saliva at day 14, suggesting that the potential for horizontal transmission of DENV-4M by bite exists (Figure 4B). Based on evidence that multiple blood feedings increase virus dissemination in *A. aegypti* (Armstrong et al., 2020), we provided a cohort of these mosquitoes a second non-infectious blood meal at 4 dpi, but this did not improve infection prevalence or intensity in this model (Supplementary Figure S1).

To test whether vertical transmission was possible, we provided ORL females fed Vero E6 P2 DENV-4M a non-infectious blood meal at 13 dpi to initiate a second gonotrophic cycle, collected and hatched eggs, and reared the resulting F1 progeny to adulthood. Since the progeny were lab reared and had no risk of acquiring DENV-4 by blood meal, we focused on female progeny due to the risk of infected females transmitting the virus to humans should they become infected by vertical transmission in the wild. Twelve pools of up to 25 surface-sterilized adult female F1s (Supplementary Table S1) were used for virus detection by rt-qPCR. Of these, only one of the 12 tested pools was positive for DENV-4, indicating that DENV-4M can undergo vertical transmission in *A. aegypti* infected *per os* (Figure 4B), but efficiency is low. We then also screened males to determine if vertical transmission was more prevalent than what we observed for females, but would otherwise be a dead-end for horizontal transmission of DENV-4M, as males do not blood feed. We noted that none of the pools of male progeny tested were positive for DENV-4M (Supplementary Table S1).

### DENV-4M Replicates in the Midgut and Ovaries of Adult Female Mosquitoes After an Infectious Blood Meal

**Figure 5:**
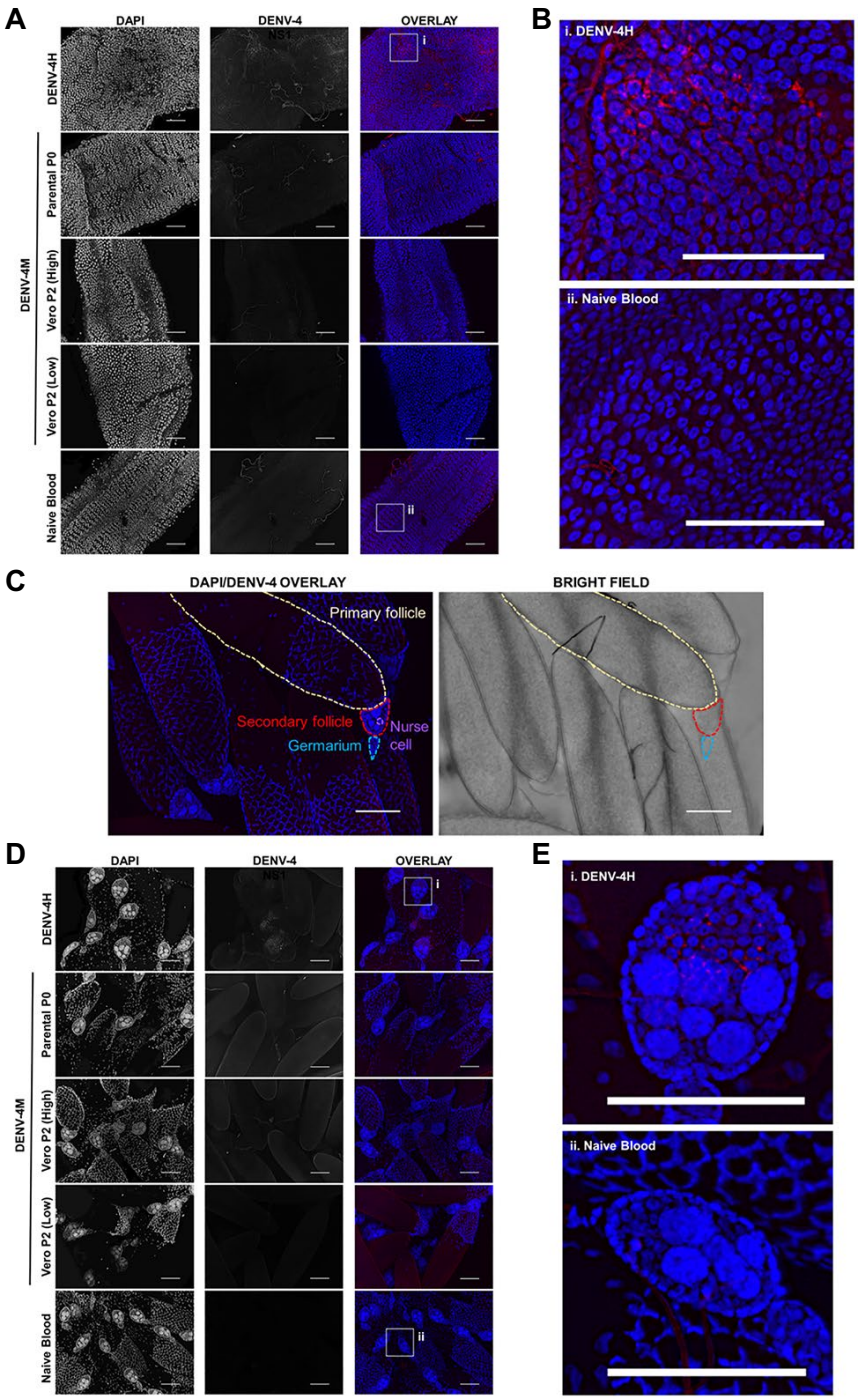
DENV-4H replicates in mosquito midguts and ovaries at 7dpi, but establishment of DENV-4M infection is delayed. Adult female ORL mosquitoes were fed a blood meal containing DENV-4H (5×10^6^ PFU/ml), Parental P0 DENV-4.M (3×10^4^ PFU/ml), Vero E6 P2 DENV-4M (high titer: 7×10^6^ PFU/ml, low titer: 4×10^3^ PFU/ml), or naive blood without virus. On day 7 post-infection, midguts **(A,B)** and ovaries **(D,E)** were dissected, and virus replication was visualized by NS1 IFA (red, middle column) with DAPI DNA counterstain (blue, left column). **(C)** Shows the major structures of a DENV-4 negative ovariole in DAPI/NS1 IFA and bright field; the primary follicle/developing embryo, the undeveloped secondary follicle containing nurse cells, and the germarium. **(B)** and **(E)** are insets chosen to show NS1 signal in virus positive conditions, compared to the naive blood negative control. Representative images were chosen from at least two independent replicates. Scale bar=100μm.

**Figure 6:**
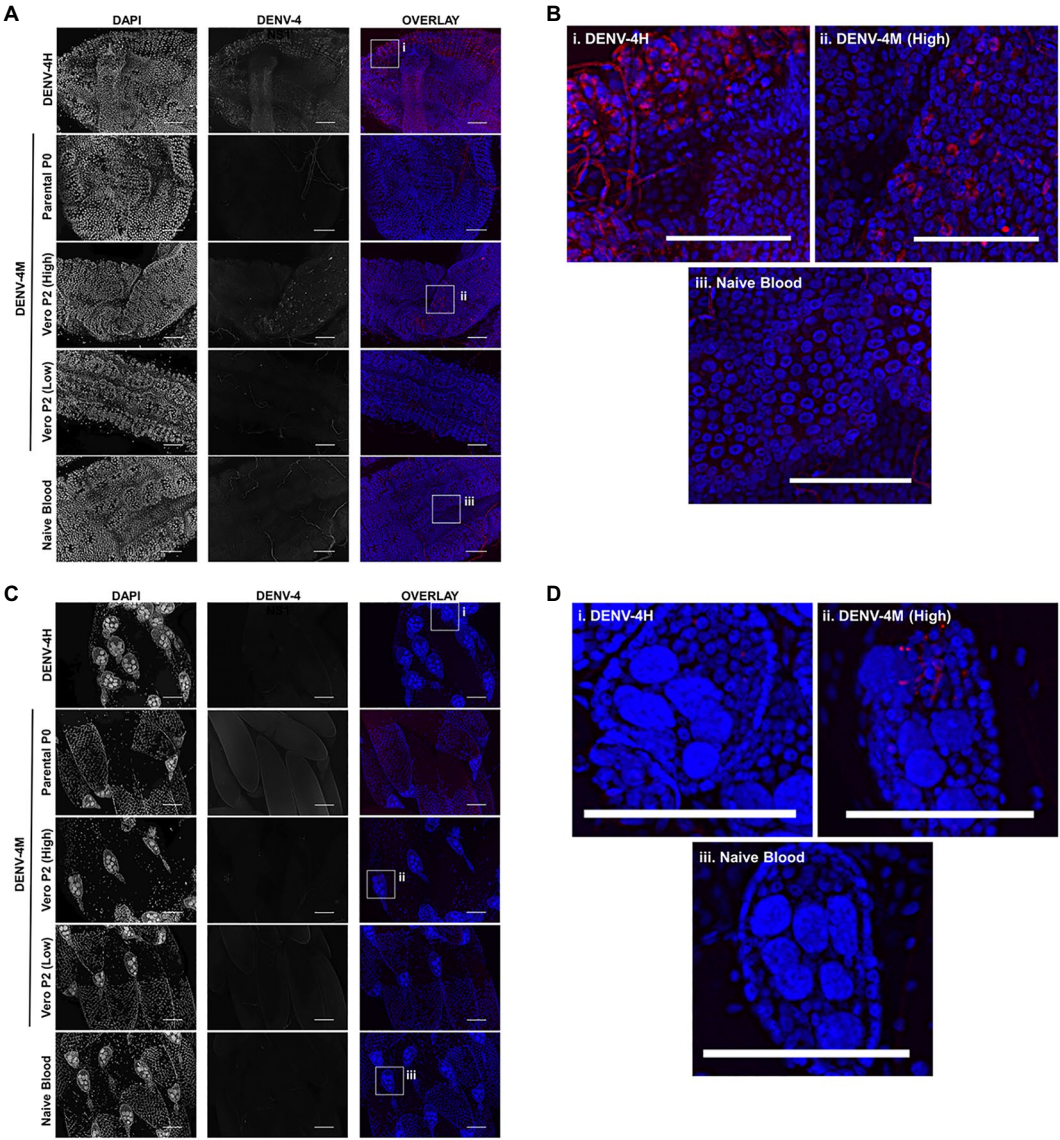
DENV-4M replicates in the midgut and ovary at 14 dpi. Adult female ORL mosquitoes were fed a blood meal containing DENV-4H (5×10^6^ PFU/ml), Parental P0 DENV-4M (3×10^4^ PFU/ml), Vero E6 P2 DENV-4M (high titer: 7×10^6^ PFU/ml, low titer: 4×10^3^ PFU/ml), or naive blood without virus. On day 14 post-infection, midguts **(A,B)** and ovaries **(C,D)** were dissected, and virus replication was visualized by NS1 IFA (red, middle column) with DAPI DNA counterstain (blue, left column). **(B)** and **(D)** are insets chosen to show the NS1 signal in virus positive conditions, compared to the naive blood negative control. Representative images were chosen from at least two independent replicates. Scale bar=100μm.

To confirm the dissemination of DENV-4M to the ovary deduced from the rt-qPCR data, as well as to compare the kinetics of this process to the wild type virus DENV-4H, adult female ORL mosquitoes were fed blood meals containing DENV-4 H, Parental P0 DENV-4M, and Vero E6 P2 DENV-4M at high titer (7×10^6^ PFU/ml) or low titer equivalent to that of the parental virus (4×10^3^ PFU/ml). At 7 and 14 dpi, midguts and ovaries were dissected from these mosquitoes, and DENV-4 replication was visualized using a DENV NS1 IFA. In both organ types, trachea displayed red autofluorescence as can be seen in the naive blood fed control panels. Image capture and analysis settings were held constant across conditions within each tissue and timepoint. At 7 dpi, only DENV-4H infection was visible in midguts (Figures 5A,B) and ovaries (Figures 5D,E). No discernable NS1 signal was seen in DENV-4M infected mosquitoes at the 7 dpi timepoint. The major structures of a DENV-4 negative ovariole are indicated in Figure 5C. At 14 dpi, DENV-4M high titer and DENV-4H produced a strong NS1 stain in the midgut (Figures 6A,B). DENV-4M high titer produced an NS1 stain in the secondary follicles of the ovaries at 14 dpi while viral replication in the ovaries of DENV-4H mosquitoes was largely abated by this timepoint (Figures 6C,D). Neither the parental P0 or the Vero E6 P2 low titer DENV-4M stocks produced noticeable NS1 stain in the ovaries or midgut at either timepoint, confirming that DENV-4M is not capable of infecting mosquitoes *per os* at the low titer, and that the infectivity difference between DENV-4M parental P0 and DENV-4M Vero E6 P2 is likely due to the different titer rather than genotypic differences between the virus stocks which arose during passage of the virus on Vero E6 cells (Figures 5, 6).

## Discussion

To gain a better estimate of mosquito transmission potential of the Manatee County, FL *Aedes aegypti* DENV-4 in the absence of directly isolated virus, we characterized an infectious clone generated from the *A. aegypti* field-derived arboviral genome sequence. Gaining retrospective insight into arbovirus transmission dynamics and disease risk in a local setting is especially important considering that the original DENV-4M virus was detected from *A. aegypti* collected from a tourist corridor in Manatee County across 2 consecutive years and in the absence of DENV-4 human index cases (Boyles et al., 2020; Mosquito-Borne Disease Surveillance, 2021).

Comparison of the DENV-4M genome sequence to a set of 234 other known dengue isolates clustered it most closely with two DENV-4 isolates from Haiti in 2014, with an estimated divergence in 2010. The next most closely related cluster was much more distantly related, with an estimated divergence in 1992 (Boyles et al., 2020). To our knowledge, no member of the DENV-4M cluster has been produced as an infectious clone before this study, and no isolates of these viruses are available to investigators. *Flavivirus* infectious clones experience sequence degradation when amplified in bacteria, and are cytotoxic to bacterial cells. This issue has been circumvented in several ways, including use of low copy plasmids (Gualano et al., 1998), use of yeast plasmids (Polo et al., 1997), use of bacterial artificial chromosomes (van der Most et al., 1999), and initial amplification of the viral genome sequence in bacteria in smaller fragments, which are ligated together to produce a full length infectious clone (Rice et al., 1989; Gallichotte et al., 2017). More recently, high fidelity polymerases have enabled the development of cell-free methods that involve amplifying genome fragments *in vitro* and ligating them into a DNA expression cassette, which can be transcribed *in vitro* or electroporated directly into vertebrate cells to obtain viable virus (Mutso et al., 2017; Amarilla et al., 2021). We utilized a high fidelity PCR and low-copy BAC plasmid to bypass cytotoxicity and genetic instability issues, as well as for speed and simplicity. However, these variations in infectious clone generation methodology do not alter the virus produced as the end product, provided the fidelity of the infectious clone to the original sequence is assured by sequencing the finished construct, as we did in this study.

The data presented here indicate that DENV-4M is a viable virus capable of replicating in insect and mammalian cell lines as well as in adult female ORL strain *A. aegypti* mosquitoes after *per os* infection. DENV is transmitted between humans when a viremic human is bitten by a competent mosquito vector. Virus in the bloodmeal infects and replicates in the mosquito midgut epithelium, then escapes and disseminates into the mosquito body cavity. To continue the transmission cycle, DENV must infect the salivary glands and escape into the saliva in the salivary gland lumen, where it will be injected into the next human host once during a subsequent bloodmeal. Tissue barriers to DENV infection exist in the mosquito, i.e., the midgut is infected but virus is not able to disseminate to other tissues, or the salivary glands are infected but no virus egresses into the salivary duct (Franz et al., 2015). Therefore, we used the midgut as a proxy for infection with DENV-4M and the saliva as a proxy for transmissibility to humans. DENV-4M is present in the midgut and the saliva in this model, posing a risk of transmission to humans by bite. It also disseminates to the ovary and is detectable in infected F1 adult female progeny, indicating vertical and transstadial transmission, which could facilitate long term maintenance within Manatee *Ae. aegypti*.

DENV-4M replication kinetics show slower and less robust replication than the wild type DENV-4H control. There was a clear dose dependent infection barrier for DENV-4M in the mosquito model as neither the parental P0 or Vero E6 P2 virus replicated in the examined tissues with the low 4×10^3^ PFU/ml titer. However, the high titer used herein is within the range of blood titers seen in viremic humans (Xu et al., 2020), so this dose-dependent infection barrier does not preclude mosquitoes from acquiring DENV-4M from infected human hosts.

Since the viral genome sequence was from nulliparous mosquitoes reared from oviposition traps and ostensibly must have undergone vertical transmission, our expectation was that it would be well adapted to replicating in insect tissue. Therefore, DENV-4M’s strong *in vitro* preference for Vero E6 cells over insect cells was surprising. However, since the virus was originally propagated in Vero E6 cells, an initial sub-selection for variants performing well in this mammalian cell line may have been inadvertently performed. This hypothesis is supported by the observation that the virus’ replication rate in insect cell lines could be markedly improved by serial passage. The sporadic nature of this adaptation to insect cell lines is illustrated by the fitness differences in virus stocks from replicate experiments, suggesting that the genetic bottleneck produced by serial passage influences the virus’ performance by inducing genetic drift, as has been previously observed in other RNA viruses (Chao, 1990; Duarte et al., 1992; Clarke et al., 1993; Lázaro et al., 2003). These results imply that minority variants, which arose randomly during passage of DENV-4M, vastly altered the virus stock’s phenotype even in only one or two passages. Genetic drift is perhaps of particular import in arboviruses since their transmission cycle requires them to maintain infectiousness in two extremely divergent hosts, and this host switching has been observed to constrain their genetic diversity (Coffey and Vignuzzi, 2011; Moutailler et al., 2011; Grubaugh et al., 2016). A comparison of the genome sequences of virus lineages derived from a single known parental genome sequence, which either succeeded or failed to adapt to the infection of mosquito cells may be a useful approach for identifying virulence factors that mediate virus infectivity for *A. aegypti* and is a compelling topic for future study of DENV-4M. The data suggest that generating infectious clones for use in arbovirus vector competence studies in insect cell lines rather than mammalian cell lines should be considered, as this may avoid a reduction in virus fitness in mosquitoes. This study demonstrated that infectious clones are extremely useful research tools, particularly to test viability when a viral genome is obtained but live virus isolation was not possible. However, when characterizing infectious clone-derived viruses, care should be taken to assess how the severe genetic bottleneck produced first by reducing the original virus population to a single consensus sequence, as well as during the cell culture process, may affect the phenotype. Taken together, these results demonstrate that our identification and sequencing of DENV-4M from mosquitoes from a central-southwest Florida county represents a *bona fide* maintenance of the virus in this environment, compelling a recalibration of the perceived risk of DENV transmission in the state.

## Data Availability Statement

The raw data supporting the conclusions of this article will be made available by the authors, without undue reservation.

## Author Contributions

XX designed and constructed the DENV-4M infectious clone and generated parental DENV-4M virus stocks. JA designed and performed mosquito and cell line infection experiments as well as the bulk of sample processing, imaging, and data analysis. XX and JA generated figures. HC and CS performed salivation assays. HC also helped perform preliminary experiments to optimize and validate IFA and imaging methods. CW contributed to processing RNA samples. P-YS and RD provided guidance on project design and writing. All authors contributed to the writing of the manuscript, but it was predominantly written by JA and XX with significant editing contributions from HC and RD. All authors contributed to the article and approved the submitted version.

## Funding

This research was supported in part by the United States Centers for Disease Control (CDC) Grant no. 1U01CK000510-03: Southeastern Regional Center of Excellence in Vector-Borne Diseases: The Gateway Program. The CDC had no role in the design of the study, the collection, analysis, and interpretation of data, or in writing the manuscript. Support was also provided by the University of Florida Emerging Pathogens Institute and the University of Florida Preeminence Initiative (RD). JA was supported as a Fellow on NIH training grant no. T32 AI 007110. P-YS was supported by NIH grant no. AI142759, AI134907, AI145617, and UL1TR001439, CDC grant for the Western Gulf Center of Excellence for Vector-Borne Diseases, and awards from the Sealy Smith Foundation, Kleberg Foundation, John S. Dunn Foundation, Amon G. Carter Foundation, Gilson Longenbaugh Foundation, and Summerfield Robert Foundation. CW was funded by the U.S. Navy and the views expressed in this manuscript are those of the author and do not necessarily reflect the official policy or position of the Department of the Navy, Department of Defense, or the U.S. Government, and is a contracted employee of the U.S. Government, this work was prepared as part of her official duties.

## Conflict of Interest

P-YS is a member of the Scientific Advisory Boards of AbImmune and is the founder of FlaviTech.

The remaining authors declare that the research was conducted in the absence of any commercial or financial relationships that could be construed as a potential conflict of interest.

P-YS laboratory has received funding support in sponsored research agreements from Pfizer, Gilead, GSK, IGM Biosciences, and Atea Pharmaceuticals for other projects unrelated to this study.

## Publisher’s Note

All claims expressed in this article are solely those of the authors and do not necessarily represent those of their affiliated organizations, or those of the publisher, the editors and the reviewers. Any product that may be evaluated in this article, or claim that may be made by its manufacturer, is not guaranteed or endorsed by the publisher.
