# Supplementary material for: Infection Kinetics and Transmissibility of a Reanimated Dengue Virus Serotype 4 Identified Originally in Wild Aedes aegypti From Florida
Source: Front Microbiol. 2021 Sep 24;12:734903. doi: 10.3389/fmicb.2021.734903 (PMC8500192; doi:10.3389/fmicb.2021.734903)
Supplement: Supplementary file 1 [file Table_1.docx]

**Supplementary Table 1. Description of vertical transmission progeny pools.** Adult F1 progeny of Vero E6 P2 DENV-4M infected females were pooled by sex and rearing container, in pools of up to 25 individuals.

| **Female Offspring Pool #** | **1** | **2** | **3** | **4** | **5** | **6** | **7** | **8** | **9** | **10** | **11** | **12** |
| --- | --- | --- | --- | --- | --- | --- | --- | --- | --- | --- | --- | --- |
| **Replicate** | 1 | 1 | 1 | 1 | 1 | 1 | 1 | 2 | 2 | 2 | 2 | 2 |
| **# Females** | 4 | 25 | 22 | 16 | 25 | 3 | 23 | 4 | 2 | 11 | 14 | 1 |
| **DENV-4 positive** | No | No | No | No | No | No | No | No | No | Yes | No | No |
| **Male Offspring Pool #** | **1** | **2** | **3** | **4** | **5** | **6** | **7** | **8** | **9** | **10** | **11** | **12** |
| **Replicate** | 1 | 1 | 1 | 1 | 1 | 2 | 2 | 2 | 2 | 2 | 2 | 2 |
| **# Females** | 7 | 2 | 11 | 7 | 17 | 1 | 24 | 25 | 9 | 25 | 25 | 11 |
| **DENV-4 positive** | No | No | No | No | No | No | No | No | No | No | No | No |
| **Male Offspring Pool #** | **13** | **14** |  |  |  |  |  |  |  |  |  |  |
| **Replicate** | 2 | 2 | 1 | 1 | 1 | 1 | 1 | 2 | 2 | 2 | 2 | 2 |
| **# Females** | 2 | 8 | 22 | 16 | 25 | 3 | 23 | 4 | 2 | 11 | 14 | 1 |
| **DENV-4 positive** | No | No | No | No | No | No | No | No | No | Yes | No | No |
