# Supplementary material for: Infection Kinetics and Transmissibility of a Reanimated Dengue Virus Serotype 4 Identified Originally in Wild Aedes aegypti From Florida
Source: Front Microbiol. 2021 Sep 24;12:734903. doi: 10.3389/fmicb.2021.734903 (PMC8500192; doi:10.3389/fmicb.2021.734903)
Supplement: Supplementary file 2 [file Table_2.DOCX]

Supplementary Table 2: Sequences of primers used in construction and sequencing of the DENV-4M infectious clone.

| **Name** | **Sequence (5′ to 3′)** | **Note** |
| --- | --- | --- |
| DV4-570V | ATGTGTGAGGACACTGTC | For sequencing |
| DV4-1154V | AAGATGTCCAACGCAAGG | For sequencing |
| DV4-2323V | AGTGTTGTGGATTGGCAC | For sequencing |
| DV4-2911V | TCACGACCAACATATGGATG | For sequencing |
| DV4-3512V | TCTGTTGTGCCTGACCTTG | For sequencing |
| DV4-4204V | ATGATGTCCCTTTAGCAGG | For sequencing |
| DV4-4801V | AGACGTTCAGGTCCTCG | For sequencing |
| DV4-5384V | TCACCGATCCTTCCAGTG | For sequencing |
| DV4-5922V | ACAAGAAGACGACCAATAC | For sequencing |
| DV4-6550V | TAGCCTTACTAGGTGCTATG | For sequencing |
| DV4-7122V | GTGAACCCAACAACCTTG | For sequencing |
| DV4-7717V | AGCATGCAGTGTCTAGAG | For sequencing |
| DV4-8801V | TCAGGAAGAACAGGGATGG | For sequencing |
| DV4-9404V | CATGGAAGTTCAGCTCATC | For sequencing |
| DV4-9998V | AGTGTGGAACAGAGTGTG | For sequencing |
| DV4-10394V | GAAGCTGTACGCGTGG | For sequencing |
| DV4-F1 | attatacgaagttatattcgatgcggccgctaatacgactcac | Forward primer PCR of F1 |
| DV4-F2 | AGGAGCTATGCATTCAGCCC | Forward primer PCR of F2 |
| DV4-F3 | GACTTACACCCCGGAGCT | Forward primer PCR of F3 |
| DV4-F4 | AGcGTCGGGAAACATCGTGAG | Forward primer PCR of F4 |
| DV4-R1 | AGCGAGGGCTGAATGCATAG | Reverse primer PCR of F1 |
| DV4-R2 | CAGCTCCGGGGTGTAAGTC | Reverse primer PCR of F2 |
| DV4-R3 | AGAGCTCACGATGTTTCCCG | Reverse primer PCR of F3 |
| DV4-R4 | gtcgactctagaggatcccac | Reverse primer PCR of F4 |
