# Supplementary material for: Infection Kinetics and Transmissibility of a Reanimated Dengue Virus Serotype 4 Identified Originally in Wild Aedes aegypti From Florida
Source: Front Microbiol. 2021 Sep 24;12:734903. doi: 10.3389/fmicb.2021.734903 (PMC8500192; doi:10.3389/fmicb.2021.734903)
Supplement: Supplementary file 3 [file Table_3.DOCX]

Supplementary Table 3. Primer sequences for detection of dengue virus serotype 4 (DENV-4) (as per Santiago et al., 2013, doi:10.1371/journal.pntd.0002311).

| **Description** | **Name** | **Sequence** | **Fluor/Quencher** | **Position** | **Product size (bp)** | **Target** |
| --- | --- | --- | --- | --- | --- | --- |
| DENV-4 forward | D4-F_CDC | TTGTCCTAATGATGCTRGTCG |  | 884-904 | 89 | DENV4 prM |
| DENV-4 reverse | D4-R_CDC | TCCACCYGAGACTCCTTCCA |  | 953-973 |  |  |
| DENV-4 probe | D4-Pr_CDC | TYCCTACYCCTACGCATCGCATTCCG | FAM/BHQ-1 | 939-965 |  |  |
