# Supplementary material for: Infection Kinetics and Transmissibility of a Reanimated Dengue Virus Serotype 4 Identified Originally in Wild Aedes aegypti From Florida
Source: Front Microbiol. 2021 Sep 24;12:734903. doi: 10.3389/fmicb.2021.734903 (PMC8500192; doi:10.3389/fmicb.2021.734903)
Supplement: Supplementary file 4 [file Presentation_1.pdf]

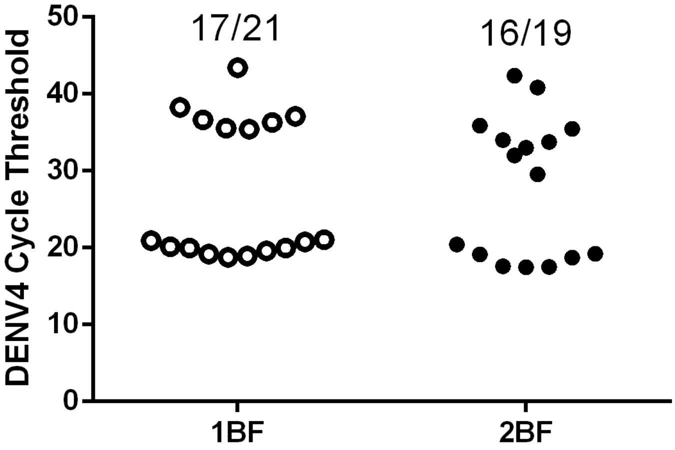

Supplementary Figure 1: Multiple blood feedings do not increase DENV-4M infection prevalence or intensity. ORL mosquitoes infected with DENV-4M Vero E6 P2 were offered a second, uninfected blood meal at 4dpi (2BF) or not (1BF). At 14dpi, whole individual mosquitoes were collected for viral genome detection by rt-qPCR. Only individuals that were positive in both rt-qPCR technical duplicates were reported as positive. The proportion of positive mosquitoes to all mosquitoes tested is above each condition on the graph.
