# Supplementary material for: Infection Kinetics and Transmissibility of a Reanimated Dengue Virus Serotype 4 Identified Originally in Wild Aedes aegypti From Florida
Source: Front Microbiol. 2021 Sep 24;12:734903. doi: 10.3389/fmicb.2021.734903 (PMC8500192; doi:10.3389/fmicb.2021.734903)
Supplement: Supplementary file 5 [file Data_Sheet_1.DOCX]

Infection kinetics and transmissibility of a reanimated dengue virus serotype 4 identified originally in wild *Aedes aegypti* from Florida– Supplementary Material

# DENV-4M Geneblock sequences

Below are the sequences of the genome fragments assembled into the DENV-4M infectious clone. The fragments are denoted F1 through F4, as illustrated in main body figure 1. Added sequences required for the cloning process are color coded for legibility. T7 promoter (red) and HDVr (blue) sequences were inserted upstream and downstream of the D4 genome. In addition, two unique restriction enzyme sites (NotI at the 5’ terminus (green) and ClaI at the 3’ terminus (pink) are added for cloning into our low-copy plasmid pCC1. Overlap sequences for ligation into the plasmid are indicated in purple. The four fragments can be assembled into a full-length genome using the Infusion cloning technologies.

>FI (1759 nt)

attatacgaagttatattcgatgcggccgctaatacgactcactatagAGTTGTTAGTCTGTGTGGACCGACAAGGACAGTTCCAAATCGGAAGCTTGCTTAACACAGTTCTAACAGTTTGTTTGAATAGAGAGCAGATCTCTGGAAAAATGAACCAACGAAAAAAGGTGGTTAGACCACCTTTCAATATGCTGAAACGCGAGAGAAACCGCGTATCAACCCCTCAAGGGTTGGTGAAGAGATTCTCAACAGGACTTTTTTCTGGGAAAGGACCCTTACGGATGGTGCTAGCATTCATCACGTTTTTGCGAGTTCTTTCCATCCCACCAACAGCAGGGATTCTGAAGAGATGGGGACAGTTGAAGAAAAATAAGGCCATTAAGATACTGACTGGATTCAGGAAGGAGATAGGCCGCATGCTGAACATCTTGAACGGGAGAAAAAGGTCAACGATAACATTGTTGTGCTTGATTCCCACCGTAATGGCGTTTCACTTGTCAACAAGAGATGGCGAACCCCTCATGATAGTGGCAAAACATGAAAGGGGGAGACCTCTCTTGTTTAAGACAACAGAGGGGATCAACAAATGCACTCTCATTGCTATGGACTTGGGTGAAATGTGTGAGGACACTGTCACGTATAAATGCCCCCTACTGGTCAATACCGAACCTGAAGACATTGATTGCTGGTGCAACCTCACGTCCACCTGGGTCATGTATGGGACATGCACCCAGAGCGGAGAACGGAGACGAGAGAAGCGCTCAGTAGCTTTGACACCACATTCAGGAATGGGATTGGAAACAAGAGCTGAGACATGGATGTCATCGGAAGGGGCTTGGAAGCATGCTCAGAGAGTAGAGAGCTGGATACTCAGAAACCCAGGATTTGCGCTCTTGGCAGGATTTATGGCTTATATGATTGGGCAAACAGGAATCCAGCGAACTGTCTTCTTTGTCCTAATGATGCTGGTCGCCCCATCCTACGGAATGCGATGCGTAGGAGTAGGAAACAGAGACTTTGTGGAAGGAGTCTCAGGTGGAGCATGGGTCGACCTGGTGCTAGAACATGGAGGATGCGTCACAACCATGGCCCAGGGAAAACCAACCTTGGATTTTGAACTGACTAAGACAACAGCTAAGGAAGTGGCTCTGTTAAGAACCTATTGTATTGAAGCCTCAATATCAAACATAACTACGGCAACAAGATGTCCAACGCAAGGAGAGCCTTATCTGAAAGAGGAACAGGACCAACAGTACATCTGCCGGAGAGATGTGGTAGACAGAGGATGGGGTAATGGCTGTGGCTTGTTTGGAAAAGGAGGAGTTGTGACATGTGCGAAGTTTTCATGTTCGGGGAAGATAACAGGCAATCTGGTCCAAATTGAGAACCTTGAATACACAGTGGTTGTAACAGTCCACAATGGAGACACCCATGCAGTAGGGAATGACACATCTAATCATGGAGTTACAGCCACGATAACTCCCAGGTCACCATCGGTAGAAGTCGAACTGCCGGACTATGGAGAACTAACACTCGATTGTGAACCCAGGTCTGGAATTGACTTTAATGAGATGATCCTAATGAAAATGAACAAGAAAACATGGCTTGTGCATAAGCAATGGTTTTTGGACCTACCTCTTCCATGGACAGCAGGAGCAGACACATCAGAGGTTCACTGGAATTACAAAGAGAGAATGGTGACATTCAAAGTTCCTCATGCTAAGAGACAGGATGTGACAGTGCTGGGATCTCAGGAAGGAGCTATGCATTCAGCCCTCGCT

>FII (3404 nt)

GAGCTATGCATTCAGCCCTCGCTGGAGCCACAGAAGTGGACTCCGGTGATGGAAATCACATGTTTGCAGGACATCTCAAGTGCAAAGTCCGTATGGAGAAATTGAGAATCAAAGGAATGTCATACACGATGTGTTCAGGAAAGTTTTCAATTGACAAAGAGATGGCAGAAACACAGCATGGGACAACAGTGGTGAAAGTCAAGTATGAAGGTGCTGGAGCTCCGTGTAAAGTCCCCATAGAGATAAGAGATGTAAACAAAGAAAAAGTGATTGGGCGCGTTATCTCATCTATCCCTTTGGCTGAGAATACCAACAGTGTGACCAACATAGAATTAGAACCCCCCTTTGGGGACAGCTACATAGTGATAGGTGTTGGAAACAGCGCATTAACACTCCATTGGTTTAGGAAAGGGAGTTCCATTGGCAAGATGTTCGAGTCCACATACAGAGGTGCAAAACGAATGGCCATTCTAGGTGAGACGGCTTGGGATTTTGGTTCTGTTGGTGGACTGTTCACATCATTGGGAAAGGCTGTGCACCAGGTTTTTGGAAGTGTGTACACAACCATGTTTGGAGGAGTCTCATGGATGATTAGAATCTTGATTGGGTTTTTAGTGTTGTGGATTGGCACGAACTCAAGGAACACTTCAATGGCCATGACGTGCATAGCTGTCGGAGGAATCACTCTGTTCTTGGGCTTCACAGTTCAAGCAGACATGGGTTGTGTGGTGTCATGGAGTGGGAGAGAGTTGAAATGTGGAAGCGGAATTTTTGTGATTGACAACGTGCACACTTGGACAGAACAGTACAAATTCCAACCAGAGTCCCCAGCGAGACTAGCGTCTGCAATATTAAATGCCCACAAAGATGGGGTCTGTGGAATTAGATCAACTACGAGGCTGGAAAATGTCATGTGGAAGCAAATAACCAATGAGCTAAACTATGTTCTCTGGGAAGGAGGACATGATCTTACTGTAGTGGCTGGGGATGTGAAGGGGGTGTTGACCAAAGGCAAGAGAGCACTCACACCCCCAGCAAGTGATCTGAAATATTCATGGAAGACATGGGGAAAAGCAAAAATCTTCACCCCTGAAGCAAGAAACAGCACATTTTTAATAGACGGACCCGATACCTCTGAATGCCCCAATGAACGAAGAGCATGGAATTCTCTTGAGGTGGAAGACTATGGATTTGGCATGTTCACGACCAACATATGGATGAAATTCCGGGAAGGAAGTTCAGAAGTGTGTGACCATAGGTTAATGTCAGCTGCAATTAAAGATCAGAAAGCTGTGCATGCTGATATGGGTTATTGGATAGAAAGCTCAAAAAACCAGACCTGGCAGATAGAGAAAGCATCTCTTATTGAAGTGAAAACATGTCTATGGCCCAAGACCCACACACTGTGGAGCAATGGAGTGCTGGAAAGCCAGATGCTCATTCCAAAATCATATGCGGGCCCTTTTTCACAGCACAACTACCGTCAGGGCTATGCCACGCAAACCGCAGGCCCATGGCACTTAGGCAAACTAGAGATAGACTTTGGAGAATGCCCCGGAACAACAGTCACAATTCAGGAGGATTGTGACCATAGAGGCCCATCTTTGAGGACCACCACTGCATCTGGAAAGTTAGTCACGCAATGGTGTTGCCGCTCCTGCACGATGCCCCCCTTAAGGTTCTTGGGAGAAGATGGGTGCTGGTATGGGATGGAGATTAGGCCCTTGAGTGAAAAAGAAGAGAATATGGTCAAATCACAGGTGGCGGCCGGACAGGGCACATCAGAGACTTTTTCTATGGGTCTGTTGTGCCTGACCTTGTTTGTGGAAGAATGCTTGAGGAGAAGAGTCACTAGGAAGCACATGATACTAGCTGTGGTGATCACTCTTTGTGCTATCATCCTGGGAGGCCTCACATGGATGGACTTGCTACGAGCCCTTATCATGTTGGGGGACACTATGTCTGGTAGAATGGGAGGACAGACCCACCTAGCCATCATGGCTGTATTCAAGATGTCACCAGGATACGTGCTGGGTGTATTTTTAAGGAAACTCACTTCAAGAGAGACAGCACTAATGGTAATAGGAATGGCCATGACAACGACACTTTCAATTCCACATGACCTTATGGAACTCATTGATGGAATATCACTAGGACTAATTTTGCTAAAAATAGTAACACAGTTTGACAACACCCAAGTGGGAACCTTAGCTCTTTCTTTGACTTTCATAAGATCAACAATGTCACTGGTCATGGCTTGGAGGACCATTATGACTGTGTTGTTCGTAGTCACACTCATTCCTTTGTGCAGGACAAGCTGTCTTCAAAAACAGTCTCACTGGGTAGAAATAACAGCACTCATCCTAGGAGCCCAAGCTCTGCCAGTGTACCTAATGACTCTTATGAAAGGGGCCTCAAAAAGATCTTGGCCTCTCAACGAGGGCATAATGGCTGTGGGTCTGGTTAGTCTCTTAGGAAGCGCTCTTTTAAAGAATGATGTCCCTTTAGCAGGCCCAATGGTAGCAGGAGGCTTACTTCTGGCGGCTTACGTGATGAGTGGCAGCTCAGCAGATCTGTCACTAGAGAAGGCCGCTAATGTGCAATGGGATGAAATGGCAGACATAACAGGCTCAAGTCCAATCATAGAAGTGAAGCAAGATGAGGATGGCTCTTTCTCCATACGAGACGTCGAGGAAACCAATATGATAACCCTTTTGGTGAAGCTGGCATTGATAACAGTGTCAGGTCTCTACCCCTTGGCAATTCCAGTCACAATGACCTTATGGTATATGTGGCAAGTGAAAACACAAAGATCAGGAGCCCTGTGGGACGTCCCCTCACCCGCTGCCACTCAAAAAGCCGCATTGTCTGAAGGAGTGTACAGGATCATGCAAAGAGGGTTATTCGGGAAAACTCAGGTTGGAGTAGGGATACATATGGAAGGTGTGTTTCACACAATGTGGCATGTTACAAGAGGATCAGTGATCTGCCACGAGACTGGGAGATTGGAGCCATCTTGGGCTGACGTCAGGAATGACATGATATCATACGGTGGGGGATGGAGGCTTGGAGACAAATGGGACAAAGAAGAAGACGTTCAGGTCCTCGCCATAGAACCAGGGAAAAATCCCAAACATGTCCAAACGAAACCAGGCCTTTTCAAGACCCTAACTGGAGAAATTGGAGCAGTAACATTAGATTTCAAACCCGGAACGTCTGGTTCTCCCATCATCAACAGGAAAGGAAAAGTCATCGGACTCTATGGAAATGGAGTGGTCACCAAATCAGGTGATTACGTTAGTGCTATAACGCAAGCCGAAAGAATTGGAGAGCCAGATTATGAAGTGGATGAGGACATTTTTCGAAAGAAAAGATTAACTATAATGGACTTACACCCCGGAGCT

>FIII (3159 nt)

GACTTACACCCCGGAGCTGGAAAGACGAAAAGAATTCTTCCATCAATAGTGAGAGAAGCCCTAAAAAGAAGGCTGCGAACTTTGATTCTGGCTCCCACGAGAGTGGTGGCGGCCGAAATGGAAGAGGCCCTACGTGGACTGCCAATCCGTTACCAGACCCCAGCTGTGAAATCAGAACACACAGGAAGAGAGATTGTAGACCTCATGTGTCATGCAACCTTCACAACAAGACTTTTGTCATCAACTAGGGTTCCAAATTACAACCTCATAGTAATGGACGAAGCACATTTCACCGATCCTTCCAGTGTCGCGGCTAGAGGATACATTTCGACCAGGGTGGAAATGGGAGAAGCAGCAGCCATCTTCATGACCGCAACCCCCCCCGGAGCGACAGATCCCTTTCCCCAGAGCAACAGCCCAATAGAAGACATCGAGAGAGAGATTCCGGAAAGGTCATGGAACACAGGGTTCGATTGGATAACAGACTATCAAGGGAAAACTGTGTGGTTTGTTCCCAGCATAAAAGCTGGAAATGACATTGCAAATTGTTTGAGAAAGTCGGGAAAGAAAGTCATTCAGTTGAGTAGGAAAACCTTTGACACAGAATATCCGAAAACGAAACTCACAGACTGGGACTTTGTGGTCACTACAGACATATCTGAAATGGGGGCTAACTTTAGAGCTGGGAGAGTGATAGACCCTAGAAGATGCCTCAAGCCAGTTATCCTAACAGATGGGCCAGAGAGAGTCATTTTAGCAGGTCCTATTCCAGTGACTCCAGCAAGCGCTGCTCAGAGAAGAGGGCGAATAGGAAGGAACCCAGCACAAGAAGACGACCAATACGTTTTCTCCGGAGACCCATTGAAAAATGATGAAGATCATGCCCACTGGACAGAAGCAAAGATGCTGCTTGACAATATCTACACCCCAGAAGGGATCATTCCAACACTGTTTGGTCCGGAAAGGGAAAAAACCCAAGCCATTGATGGAGAGTTTCGTCTCAGAGGGGAACAAAGGAAGACTTTTGTGGAATTAATGAGGAGAGGAGACCTTCCAGTGTGGCTGAGCTATAAGGTAGCATCTGCTGGCATTTCTTACAAAGATCGGGAATGGTGCTTCACTGGGGAAAGAAACAATCAAATTTTAGAAGAAAACATGGAGGTTGAAATTTGGACTAGAGAGGGGGAAAAGAAAAAACTGAGGCCAAGATGGTTAGATGCACGTGTATACGCTGACCCCATGGCTTTGAAGGATTTCAAGGAGTTTGCCAGTGGAAGGAAGAGTTTGACTCTCGACATTCTAACAGAGATTGCCAGTTTGCCAACTTATCTTTCCTCTAGGGCCAAGCTCGCCCTTGATAACATAGTCATGCTCCACACAACAGAAAGAGGAGGGAGGGCCTATCAACATGCCCTGAACGAACTTCCGGAGTCACTGGAAACACTCATGCTTGTAGCCTTACTAGGTGCTATGACAGCAGGCATCTTCCTGTTTTTCATGCAAGGGAAAGGAATAGGGAAATTGTCAATGGGTTTGATAACCATTGCGGTGGCTAGTGGCTTGCTCTGGGTAGCAGAAATTCAACCACAGTGGATAGCGGCCTCAATCATACTGGAATTTTTTCTCATGGTACTGTTGATACCAGAACCAGAAAAACAAAGGACCCCACAAGACAATCAATTGATCTACGTCATATTGGCCATTCTCACCATTATAGGTCTAATAGCAGCCAACGAGATGGGGCTGATAGAAAAAACAAAAACGGACTTTGGGTTTTACCAGGTAAAGACAGAAACCACCATCCTCGATGTGGACTTGAGACCAGCTTCGGCATGGACGCTCTATGCGGTAGCCACCACAATTCTGACTCCCATGCTGAGACACACCATAGAAAACACATCGGCCAATCTATCTTTAGCAGCCATTGCCAACCAGGCAGCCGTCCTAATGGGGCTTGGAAAAGGATGGCCGCTCCACAGAATGGACCTCGGTGTGCCGCTGTTGGCAATGGGATGCTATTCTCAAGTGAACCCAACAACCTTGATAGCATCCTTAGTCATGCTTTTAGTCCATTATGCAATAATAGGCCCAGGATTGCAGGCAAAAGCCACAAGAGAGGCCCAGAAAAGGACAGCTGCTGGAATCATGAAAAATCCCACAGTGGACGGGATAACAGTAATAGATCTAGAGCCAATATCCTATGACCCAAAATTTGAAAAGCAATTAGGGCAGGTCATGCTACTAGTCTTGTGTGCTGGACAACTACTCCTGATGAGAACAACATGGGCTTTCTGTGAAGTCTTGACTTTGGCCACAGGACCAATCTTGACCTTGTGGGAGGGCAACCCGGGAAGGTTTTGGAACACGACCATAGCCGTATCCACAGCCAACATTTTCAGGGGAAGTTACTTAGCGGGAGCTGGACTGGCTTTTTCACTCATAAAGAATGCACAAACCCCTAGGAGGGGAACTGGGACCACAGGAGAGACACTGGGAGAGAAGTGGAAGAGACAGCTAAACTCATTAGACAGAAAAGAGTTTGAAGAATATAAAAGAAGTGGAATACTGGAAGTGGATAGGACTGAAGCTAAGTCTGCCCTGAAAGATGGGTCTAAAATCAAGCATGCAGTGTCTAGAGGGTCCAGTAAGATCAGATGGATTGTTGAGAGAGGGATGGTAAAACCAAAAGGGAAAGTTGTAGATCTTGGCTGTGGGAGAGGAGGATGGTCTTATTACATGGCGACACTCAAGAACGTGACTGAAGTGAAAGGGTATACAAAAGGAGGTCCAGGACATGAAGAACCGATACCTATGGCTACTTATGGCTGGAATTTGGTCAAACTCCATTCAGGGGTTGACGTGTTCTATAAACCCACAGAGCAAGTGGACACCCTGCTCTGTGATATTGGGGAGTCATCTTCTAATCCAACAATAGAGGAAGGAAGAACATTAAGAGTTCTGAAGATGGTGGAGCCATGGCTCTCTTCAAAACCAGAATTCTGCATAAAAGTCCTTAACCCCTACATGCCAACAGTCATAGAAGAGCTAGAGAAACTGCAGAGGAAACATGGTGGGAACCTTGTCAGATGCCCGCTGTCCAGGAACTCCACCCATGAGATGTATTGGGTGTCAGGAGCGTCGGGAAACATCGTGAGCTCT

>FIV (2510 nt)

GTCGGGAAACATCGTGAGCTCTGTGAACACAACATCAAAGATGTTGTTGAACAGATTCACAACAAGGCATAGGAAACCCACTTATGAGAAGGACGTAGATCTTGGGGCAGGAACGAGAAGTGTCTCTACTGAAACAGAAAAACCAGACATGACAATCATTGGGAGAAGGCTTCAGCGATTGCAAGAAGAGCACAAAGAAACATGGCATTATGACCAGGAAAACCCATACAGAACCTGGGCGTATCATGGAAGCTATGAAGCTCCTTCGACAGGCTCTGCATCCTCTATGGTGAACGGGGTGGTGAAACTGCTAACAAAACCCTGGGATGTGATTCCAATGGTGACTCAGTTAGCCATGACAGATACAACCCCTTTTGGGCAACAAAGAGTGTTCAAAGAGAAGGTGGATACCAGAACACCGCAACCAAAACCAGGCACACGAATGGTTATGACCACGACAGCCAATTGGCTATGGGCCCTCCTTGGAAAGAAGAAGAATCCCAGATTGTGCACAAAGGAAGAGTTCATCTCAAAAGTTAGAGCAAACGCAGCCATAGGCGCGGTCTTTCAGGAAGAACAGGGATGGACATCAGCCAGTGAAGCTGTGAATGACAGCCGGTTTTGGGAGCTGGTTGACAAAGAAAGGGCCCTTCACCAGGAAGGGAAATGTGAATCGTGTGTCTACAACATGATGGGAAAACGTGAGAAAAAGTTAGGAGAGTTTGGCAGAGCCAAGGGAAGCCGAGCAATCTGGTACATGTGGCTGGGAGCTCGGTTTCTGGAATTTGAAGCCCTGGGTTTTTTGAATGAAGACCACTGGTTTGGCAGAGAAAATTCATTGAGTGGAGTGGAAGGGGAAGGTCTGCACAGATTGGGATACATCTTGGAGGAGATAGACAAGAAGGATGGAGACCTAATGTATGCTGACGACACAGCAGGCTGGGACACAAGAATCACTGAGGATGACCTTCAAAATGAGGAACTGATCACGGAACAGATGGCTCCCCACCATAAGATCCTAGCCAAAGCTATTTTCAAACTAACCTATCAGAACAAAGTGGTGAAAGTCCTCAGACCCACACCGAGAGGAGCGGTGATGGACATCATTTCCAGGAAAGACCAAAGAGGTAGTGGACAAGTTGGAACATATGGTTTGAACACATTCACCAACATGGAAGTTCAGCTCATCCGCCAAATGGAAGCTGAAGGAGTCATCACACAAGATGACATGCAGAACCCAAAAGGGTTGAAAGAAAGGGTTGAGAAATGGCTGAGAGAGTGTGGTGTCGACAGGTTAAAAAGGATGGCAATCAGTGGAGACGATTGCGTGGTGAAGCCCCTAGATGAGAGATTTGCCACTTCCCTCCTCTTCTTGAACGACATGGGAAAGGTGAGGAAAGACATACCGCAGTGGGAACCATCTAAGGGATGGAAAAACTGGCAAGAGGTGCCTTTTTGCTCCCACCACTTTCACAAAATCTTTATGAAGGATGGCCGCTCACTAGTAGTTCCATGTAGAAACCAGGATGAACTGATAGGGAGAGCTAGAATCTCGCAGGGGGCTGGATGGAGCTTGAGAGAAACAGCCTGCCTGGCCAAAGCTTACGCCCAGATGTGGTCGCTTATGTACTTCCACAGAAGGGATCTGCGTTTAGCCTCCATGGCCATATGCTCAGCAGTTCCAACGGAATGGTTTCCAACAAGCAGAACAACATGGTCAATCCACGCTCATCACCAATGGATGACCACTGAAGACATGCTCAAAGTGTGGAACAGAGTGTGGATAGAGGACAACCCCAATATGATTGACAAGACTCCAGTCCATTCGTGGGAAGATATACCTTACCTAGGGAAAAGAGAGGATTTATGGTGTGGATCCTTGATTGGACTTTCTTCTAGAGCCACCTGGGCGAAGAACATTCACACGGCCATAACTCAGGTCAGGAACCTGATCGGAAAAGAGGAATACATGGATTACATGCCAGTAATGAGAAGATACAGTGCTCCTTCAGAGAGTGAAGGAGTTCTGTAATCACCAACAACAAATACCAAAGGCTATTGAAGTCAGGCCACTTGTGCCACGGCTTGAGCAAACCGTGCTGCCTGTAGCTCCGCCAACAATGGGAGGCGTAATAATCCCTAGGGAGGCCATGCGCCACGGAAGCTGTACGCGTGGCATATTGGACTAGCGGTTAGAGGAGACCCCTCCCACCACTGACAAAACGCAGCAAAAAGGGGGCCCGATGCCAGGAGGAAGCTGTACTCCTGGTGGAAGGACTAGAGGTTAGAGGAGACCCCCCCAACACAAAAACAGCATATTGACGCTGGGAAAGACCAGAGATCCTGCTGTCTCTACAACATCAATCCAGGCACAGAGCGCCGCAAGATGGATTGGTGTTGTTGATCCAACAGGTTCTGGGTCGGCATGGCATCTCCACCTCCTCGCGGTCCGACCTGGGCTACTTCGGTAGGCTAAGGGAGAAGAATCGATgtgggatcctctagagtcgac
